# Relationship of thyroid parameters with chronic inflammation in patients with euthyroid type 2 diabetes

**DOI:** 10.3389/fendo.2024.1433782

**Published:** 2024-10-25

**Authors:** Wei Shi, Dan Chen, Wei Chen, Yulu Chen, Xiaoling Fu, Yong Xiao, Wei Duan, Jing Zhang

**Affiliations:** Department of Endocrinology, Hubei Integrated Traditional Chinese and Western Medicine Hospital, Hubei University of Chinese Medicine, Wuhan, China

**Keywords:** thyroid hormone, thyroid hormone sensitivity, euthyroid, diabetes, inflammation

## Abstract

**Aims:**

We evaluated the association of multiple thyroid parameters with the levels interleukin 6 (IL6) and interleukin 8 (IL8) in patients with euthyroid type 2 diabetes (T2D).

**Methods:**

A total of 166 adults with euthyroid T2D were examined. Serum IL6, IL8, triiodothyronine (FT3), free thyroxin (FT4), thyroid-stimulating hormone (TSH), five thyroid sensitivity indexes (FT3/FT4, TSH index [TSHI], thyrotroph T4 resistance index [TT4RI], thyroid feedback quantile-based index by FT3 [TFQI_FT3_], and TFQI_FT4_), and biochemical parameters were determined.

**Results:**

The median age was 64 years (IQR: 54.75,70) and the median duration of diabetes was 10 years (IQR: 3,18). Patients with high levels of IL6 (≥2.38 pg/mL) had lower levels of FT3 and TFQI_FT3_ (*P*<0.05). Patients with high levels of IL8 (≥18.1 pg/mL) had lower levels of TSH and higher levels of FT4 (*P*<0.05). IL6 was negatively correlated with FT3 (r=−0.359, *P*<0.001), TFQI_FT3_ (r=−0.273, *P*=0.009), and FT3/FT4 (r=−0.22, *P*=0.037). IL8 was negatively correlated with TSH (r=−0.256, *P*=0.01), TSHI (r=−0.226, *P*=0.033), and TT4RI (r=−0.244, *P*=0.021). Binary logistic regression analysis with multiple adjusted models showed that a high level of IL6 was negatively associated with FT3 (aOR: 0.529; 95%CI: 0.302, 0.926, *P*=0.026), and that a high level of IL8 was negatively associated with TSH (aOR: 0.343; 95%CI: 0.155, 0.759; *P*=0.008) and TT4RI (aOR: 0.398;95%CI: 0.191, 0.831; *P=*0.014).

**Conclusion:**

Patients with euthyroid T2D who had lower levels of FT3 had significantly higher levels of IL6, and those with lower levels of TSH and TT4RI had significantly higher levels of IL8.

## Introduction

The International Diabetes Federation (IDF) recently estimated the global prevalence of diabetes was 10.5% among adults (1). The estimated prevalence of diabetes in China is 11.2%,so China has the greatest number of people with diabetes (2). The annual diabetes-related health expenditures are also considerable, and were estimated at US$966 billion globally and US$165.3 billion in China, ranking second worldwide (1). Although there are numerous treatments for diabetes, their effectiveness is limited, at least partly because of the complex pathogenesis of this disease. Consequently, it is imperative to elucidate the risk factors associated with diabetes and examine their effects on the pathogenesis of this disease.

Thyroid hormones and thyroid-sensitive biomarkers have significant associations with metabolic disorders, particularly diabetes. Specifically, deviations in thyroid hormone levels-increased or decreased-and abnormalities in thyroid-sensitive indicators are closely associated with diabetes-related endpoint events. Notably, this correlation persists even among individuals with normal thyroid function (3–8). Similarly, our prior research identified an association between FT3 and chronic kidney disease (CKD) in euthyroid individuals with diabetes (9). Nevertheless, the underlying mechanisms governing the relationship between thyroid parameters and diabetes and diabetes-related endpoint events remain unclear.

Chronic low-grade inflammation is widely recognized as a significant contributor to the pathogenesis of diabetes (10). Specifically, the levels of interleukin 6 (IL6) and IL8 have strong correlations with the level of glycated hemoglobin (HbA1c) and with subsequent cardiovascular and renal complications in individuals with type 2 diabetes (T2D) (11–13). The levels of IL6 and IL8 are also positively associated with thyroid hormone levels in patients with thyroid disorders (14).

Several studies with small sample sizes have examined the correlation between thyroid hormones and inflammation markers in diabetes, but the results have been inconsistent. For example, a cross-sectional study of 278 patients with T2DM demonstrated positive correlations of TSH, FT3, FT4, and various markers of chronic inflammation (4). Another observational study focused on patients with non-thyroidal illnesses, including a subset of patients with diabetes, and demonstrated that only FT3 and IL-6 had positive relationships (15). Furthermore, in patients with euthyroid diabetes, the standard thyroid sensitivity indices (crucial metrics for assessing the relationship between the thyroid system and diabetes) have not yet been investigated in relation to chronic inflammation. Thus, the present study investigated the association of various thyroid parameters, including thyroid hormones and indexes of central and peripheral sensitivity to thyroid hormones, with inflammation markers in patients with euthyroid T2D.

## Materials and methods

### Study subjects

This cross-sectional study was conducted at the Inpatient Department of Hubei Integrated Traditional Chinese Medicine (TCM) and Western Medicine (WM) Hospital in Wuhan, China. A total of 182 consecutive adult patients diagnosed with euthyroid T2D were initially examined from July 2021 to October 2022, and 166 of these patients were finally included. Participants were excluded if they had any of the following criteria: (*i*) history of thyroid surgery or treatment for a thyroid disease; (*ii*) acute complications of T2D (e.g., diabetic ketoacidosis, hypertonic coma, foot ulcers); (*iii*) chronic liver disease, undergoing dialysis, or a prior kidney transplant; (*iv*) pregnancy; (*v*) abnormal thyroid function based on deviations from the normal reference ranges for thyroid stimulating hormone (TSH, 0.35–5.5 μIU/mL), free thyroxine (FT4, 11.5–22.7 pmol/L), and tri-iodothyronine (FT3, 3.5–6.5 pmol/L); (*vi*)history of any illness associated with inflammatory or autoimmune disorders; and (*vii*) missing data.Ethical approval for this study protocol was obtained from the Ethical Research Committee of Hubei Integrated TCM and WM Hospital (No.2021-033).

### Clinical examination and laboratory measurements

A physician interviewed all subjects to record medical histories, and a trained examiner measured body weight. Smokers were defined as those who smoked at least 1 cigarette per day continuously for 6 months. Alcohol drinkers were those who consumed at least 20 g of alcohol per day during the previous 1 month. The Medical Examination Center at Hubei Integrated TCM and WM Hospital analyzed all blood and urine samples. Our previous publication (9) described the methods and instruments used to measure biochemical parameters, including blood urea nitrogen (BUN), serum creatinine (SCr), alanine aminotransferase (ALT), aspartate aminotransferase (AST), triglycerides (TG),total cholesterol (TC), high-density-lipoprotein cholesterol(HDL-C),low-density-lipoprotein cholesterol (LDL-C),serum cystatin C (Cys C), blood glucose, hemoglobin A1c (HbA1c), urine albumin/creatinine ratio (UACR),TSH,FT4,and FT3.Chemiluminescence assays were used to measure the serum level of 25-hydroxy vitamin D ([25(OH)D], ADVIA Centaur XPT, Siemens Healthcare Diagnostics Inc., NY,USA) and the plasma levels IL6 and IL8 (IMMULITE 1000, Siemens Healthcare Diagnostics Inc., NY,USA).

### Definitions and clinical indexes

Hypertension was defined as a systolic blood pressure of at least 140 mmHg and/or a diastolic blood pressure of at least 90 mmHg, or by use of an antihypertensive drug. The triglyceride-glucose (TyG) index (16) was calculated as:


(1) Ln([fasting triglycerides, mg/dL] × [fasting glucose, mg/dL]/2)

The thyroid sensitivity indices included four central indexes and one peripheral index. The four central indexes were the TSH index [TSHI (17)], thyrotroph T4 resistance index [TT4RI (18)], thyroid feedback quantile-based FT3 [TFQI_FT3_ (19)], and TFQI_FT4_ (20):


(2) TSHI= Ln([TSH, mIU/L] + 0.1345 × [FT4,pmol/L])

(3) TT4RI= (FT4, pmol/L) × (TSH, mIU/L)

(4) $${\text{TFQI}}_{\text{FT}3}=\text{ cumulative distribution function }\left(\text{cdfFT}3\right)\;–\;\left(1\;–\text{ cdfTSH}\right)$$

(5) $${\text{TFQI}}_{\text{FT}4}=\text{cumulative distribution function }\left(\text{cdfFT}4\right)\;–\;\left(1\;–\text{ cdfTSH}\right)$$

For TSHI and TT4RI, a lower value indicated greater sensitivity; for TFQI_FT3_ and TFQI_FT4_, a negative value indicated greater sensitivity (3, 19).

The peripheral index (FT3/FT4 was:


(6) FT3/FT4 = (FT3, pmol/L)/(FT4, pmol/L)

A higher FT3/FT4 value indicated greater sensitivity (3).

### Statistical analysis

SPSS version 22.0 (SPSS, Chicago, IL, USA) was used for statistical analyses. Data are presented as means ± standard deviations (SDs), medians (interquartile ranges [IQRs]), or numbers (percentages), as appropriate. For comparisons, participants were divided into two groups based on the median level of IL6 (2.38 pg/mL) and based on the median level of IL8 (18.1 pg/mL). These groups were then compared using Student’s *t*-test or the Mann-Whitney U test for continuous variables, or the Chi-squared test for categorical variables. Spearman’s correlation analysis was employed to evaluate the univariate correlations of IL6 and IL8 with thyroid parameters and various clinical characteristics.

Binary logistic regression was utilized to estimate the odds ratio (OR) of high IL6 (≥2.38 pg/mL) and high IL8(≥18.1pg/mL) for 1 SD change of the indices of thyroid hormone and thyroid hormone sensitivity in both adjusted models. In this analysis, continuous variables with non-normal distributions were log-transformed before calculation of OR values. Model 1 adjusted for sex, age, weight, Log (diabetes duration), Log (HbA1c), smoking, drinking, hypertension, coronary heart disease (CHD), use of a renin angiotensin system inhibitor (RASI), use of a sodium-glucose transport protein inhibitor (SGLT2I), use of a glucogon-like peptide-1 receptor agonist (GLP1RA), and use of a lipid-lowering treatment. Model 2 adjusted for all Model 1 factors and also for use of metformin, estimated glomerular filtration rate (eGFR), 25(OH)D, Log (UACR), Cys C, Log (TyG index), Log (AST/ALT), and Log (IL8). Model 3 adjusted for all Model 1 factors and also for use of metformin, eGFR, 25(OH)D, Log (UACR), Log (Cys C), Log (TyG index), Log (AST/ALT), and Log (IL6). A p-value below 0.05 was considered significant in all analyses.

## Results

We analyzed 166 adult patients who were diagnosed with euthyroid T2D. The median age was 64 years (IQR: 54.75,70),46.2% of the patients were female, the median duration of T2D was 10 years (IQR: 3,18), and the median level of HbA1c was 8.8% (IQR: 7.18,10.6). We divided these patients into two groups according to the median levels of two markers of chronic inflammation: IL6 and IL8 (**Table 1**). Compared with the low IL6 group (<2.38pg/mL), the high IL6 group had significantly higher levels of SCr, UACR, Cys C, and AST/ALT and significantly lower levels of FT3, TFQI_FT3_, ALT, 25(OH)D, and eGFR (all *P*<0.05).Compared with the low IL8 group (<18.1pg/mL), the high IL-8 group had significantly higher levels of FT4 and IL-6, and significantly less usage of GLP1RAs (all *P*<0.05). In addition, the serum level of TT4RI in high IL8 group tended to be lower than in the low IL8 group (P=0.054). These two groups had no statistically significant differences in gender, history of hypertension, history of CHD, smoking, alcohol consumption, use of lipid-lowering agents, use of antihypertensive medications, and (except for GLP1RA) use of treatments for T2D (all P>0.05).

**Table 1:** Baseline characteristics of the study population according to the levels of IL6 and IL8^†^.

Variable	Low IL6 (<2.38pg/mL) (n=81)	High IL6 (≥2.38pg/mL) (n=85)	Low IL8 (<18.1pg/mL) (n=82)	High IL8 (≥18.1pg/mL) (n=84)
Age (years)	64 (49,67)	65 (56,69)	57 (45.5,66)	62.5 (56,67)
Gender (M/F)	46/35	50/35	46/36	48/36
Smoking	23/81	20/85	20/82	20/84
Drinking	14/81	18/85	17/82	15/84
T2D duration (years)	9 (0.5,16)	10 (5,19)	6 (0.019,17)	8 (3,14.5)
Weight (kg)	70 (62,77)	70 (60,80)	70.5 (60,78.5)	70 (63,78.25)
FPG (mmol/L)	7.105 (5.825,8.82)	7.58 (6.07,9.98	7.69 (6.24,9.305)	7.28 (5.98,8.665)
PG2H (mmol/L)	17.284 ± 4.926	17.337 ± 5.802	17.190 ± 5.686	17.393 ± 5.169
HbA1_C_ (%)	9.5 (7.5,10.8)	9 (7.6,11)	10.55 (8.05,11.85)	9.5 (7.55,10.75)
TG (mmol/L)	1.51 (1.16,2.765)	1.68 (1.25,2.52)	1.61 (1.18,2.425)	1.57 (1.19,2.515)
TC (mmol/L)	4.703 ± 1.197	4.758 ± 1.438	4.914 ± 1.422	4.503 ± 1.227
LDL (mmol/L)	1.915 (1.445,2.23)	2.13 (1.6,2.56)	2.04 (1.5,2.3)	1.81 (1.315,2.23)
HDL (mmol/L)	1.19 (1.075,1.38)	1.26 (1.06,1.68)	1.14 (0.975,1.305)	1.26 (1.095,1.43)
TSH (µIU/mL)^#^	1.845 (1.37,2.716)	1.735 (1.345,2.501)	2.2635 (1.426,3.937)	1.558 (1.124,2.125)
FT3 (pmol/L)^&^	4.827 ± 0.52	4.462 ± 0.539	4.641 ± 0.604	4.620 ± 0.504
FT4 (pmol/L)^#^	16.198 ± 2.372	15.759 ± 1.647	15.639 ± 2.044	16.194 ± 2.001
BUN (mmol/L)	5.31 (4.465,6.25)	5.39 (4.54,7.38)	5.61 (4.67,5.88)	5.4 (4.34,7.89)
SCr (µmol/L)^*^	59.9 (51.6,72.7)	73.6 (51.5,95.1)	70.75 (56.45,89.45)	58.4 (48.8,78)
ALT (U/L)^&^	15.5 (10,31)	11 (7,19)	13 (6.5,22)	11 (8,19)
AST (U/L)	18.5 (15.5,30.5)	19 (15,22)	18 (15,22.5)	18.5 (12,25)
AST/ALT^&^	1.3077 (0.860,1.996	1.7098 (1.1835,2.4857)	1.4143 (1,2.5)	1.5 (1,2)
eGFR (mL/min/1.73 m^2^)^&^	120.369 ± 35.023	92.959 ± 40.619	104.027 ± 43.751	108.772 ± 37.602
Cys C (mg/dL)^*^	0.8 (0.69,0.995)	0.89 (0.76,1.13)	0.955 (0.74,1.215)	0.865 (0.72,1.12)
UACR (mg/g)^*^	28.79 (13.9,52.3)	47.82 (15.99,309.74)	30.03 (13.51,116.36)	32.91 (14.79,198.65)
25 (OH)D (ng/mL)^*^	21.962 ± 7.316	18.366 ± 7.902	21.147 ± 7.726	18.930 ± 7.678
TyG Index	9.752 (9.474,10.368)	10.025 (9.59,10.56)	9.839 (9.567,10.56)	9.892 (9.639,10.416)
TSHI	2.84 (2.51,3.14)	2.71 (2.35,3.22)	2.97 (2.51,3.33)	2.645 (2.23,2.93)
TT4RI	29.4 (21.78,44.18)	27.34 (22.56,42.47)	35 (22.83,61.22)	24.525 (20.57,32.14)
TFQI_FT3_^*^	0.117 ± 0.241	−0.006 ± 0.288	0.099 ± 0.286	0.013 ± 0.252
TFQI_FT4_	0.030 ± 0.271	−0.023 ± 0.266	0.019 ± 0.271	−0.016 ± 0.266
FT3/FT4	0.3 (0.265,0.335)	0.28 (0.26,0.3)	0.3 (0.27,0.34)	0.28 (0.25,0.31)
IL6 (pg/mL)^&#^	1.0 (0.5, 2.03)	3.33 (2.85, 5.63)	2.02 (0.7,2.75)	2.63 (1.0,3.58)
IL8 (pg/mL)^§^	16.8 (13.15,25.2)	21.8 (13.1,45.6)	12.85 (9.04,14.8)	32.5 (23.2,85.45)
Hypertension	36/81	51/85	37/82	49/84
CHD	7/81	14/85	8/82	12/84
Lipid-lowering treatment	22/81	14/85	17/82	17/84
Diabetes treatment							
Glp-1RA^#^	7/81	6/85	10/82	2/84
SGLT-2i	13/81	8/85	10/82	10/84
Metformin	38/81	31/85	33/82	34/84
Insulin	21/81	20/85	15/82	21/84
Dpp-4i	17/81	20/85	19/82	18/56
Alpha-glucosidase inhibitor	33/81	32/85	37/82	28/84
Sulfonylureas	20/81	19/85	14/82	24/84
Thiazolidinediones	21/81	6/85	12/82	8/84
Anti-hypertensive agents							
RASi	22/81	19/85	17/82	25/84
CCB	23/81	32/85	27/82	28/84

^†^Variables are presented as count/total if categorical, and mean ± standard deviation or median (IQR) if continuous.

Significance of intergroup differences is indicated by *(IL6: P<0.05), ^&^(IL6: P<0.01), ^#^(IL8: P<0.05), and ^§^(IL8: P<0.01).

FPG, fasting plasma glucose; PG2H, 2-h postprandial blood glucose; HbA1c, glycated hemoglobin; TG, triglyceride; TC, total cholesterol; LDL-C, low-density lipoprotein-cholesterol; HDL-C, high-density lipoprotein-cholesterol; TSH, thyroid stimulating hormone; FT3, free triiodothyronine; FT4, free thyroxine; TSHI, TSH index; TT4RI, TSH T4 resistance index; TFQI_FT3_, the thyroid feedback quantile-based index calculated by FT3; TFQI_FT4_, the thyroid feedback quantile-based index calculated by FT4;BUN, blood urea nitrogen; Scr, serum creatinine; TyG Index, triglyceride-glucose index; UUACR, urinary albumin-to creatinine ratio; ALT, alanine transaminase; AST, aspartic transaminase;Cys-C, cystatin C; eGFR, estimated glomerular filtration rate; IL-6, Interleukin-6; IL-8, Interleukin -8; PTH, parathyroid hormone;25 (OH)D, 25-HydroxyvitaminD; CHD, coronary heart disease;Glp-1RA, glucagon-likepeptide-1 receptor agonists; SGLT-2i, sodium/glucose contransporter 2 inhibitors; DPP-IVi, dipeptidypeptidase-4 inhibitors; RASi, rennin-angiotensin system inhibitor; CCB, Calcium-channel antagonist.

We then used Spearman correlation analysis to evaluate relationships of IL-6 and IL-8 with thyroid parameters and clinical characteristics (**Table 2**). Among the thyroid parameters, IL-6 had significant negative correlations with FT3 (r=−0.359, *P*<0.001), TFQI_FT3_(r = −0.273, *P*=0.009), and FT3/FT4 (r=−0.22, *P*=0.037). IL-8 had significant negative correlations with TSH (r=−0.256, *P*=0.015), TSHI (r=−0.226, *P*=0.033), and TT4Ri (r=−0.244, *P*=0.021). Among the clinical variables, IL6 had positive correlations with age (r=0.221, *P*=0.018), duration of diabetes (r=0.212, *P*=0.024), Cys C (r=0.299, *P*=0.002), and AST/ALT (r=0.314, *P*<0.001) and significant negative correlations with eGFR (r=−0.337, *P*<0.001) and 25(OH)D (r=−0.22, *P*=0.023). IL8 had no significant correlations with any of the clinical variables (all *P*>0.05).

**Table 2:** Correlation of the levels of IL6 and IL8 with thyroid parameters and clinical characteristics.

Variable	IL6 (pg/mL)		IL8 (pg/mL)	
	r	P value	r	P value
TSH (µIU/mL)	−0.026	0.806	−0.256	0.015
FT3 (pmol/L)	−0.359	<0.001	−0.014	0.896
FT4 (pmol/L)	−0.133	0.211	0.045	0.676
TSHI	−0.075	0.48	−0.226	0.033
TT4RI	−0.042	0.691	−0.244	0.021
TFQI_FT3_	−0.273	0.009	−0.194	0.069
TFQI_FT4_	−0.144	0.176	−0.156	0.144
FT3/FT4	−0.22	0.037	−0.063	0.560
Age (years)	0.221	0.018	0.141	0.139
Weight (kg)	−0.22	0.828	−0.028	0.781
Diabetes duration (years)	0.212	0.024	0.136	0.156
HbA1c (%)	−0.024	0.803	−0.056	0.565
eGFR (mL/min/1.73 m^2^)	−0.337	<0.001	0.184	0.063
Cys C (mg/dL)	0.299	0.002	−0.079	0.422
UACR (mg/g)	0.149	0.126	−0.073	0.462
25(OH)D (ng/mL)	−0.22	0.023	−0.108	0.278
TyG Index	−0.005	0.960	−0.054	0.584
AST/ALT	0.314	<0.001	0.000	0.998

TSH, thyroid stimulating hormone; FT3, free triiodothyronine; FT4, free thyroxine; TSHI, TSH index; TT4RI, TSH T4 resistance index; TFQIFT3, the thyroid feedback quantile-based index calculated by FT3; TFQIFT4, the thyroid feedback quantile-based index calculated by FT4; HbA1c, glycated hemoglobin; eGFR, estimated glomerular filtration rate; Cys-C, cystatin C; UUACR, urinary albumin-to creatinine ratio;25(OH)D, 25-Hydroxyvitamin D; TyG index, triglyceride-glucose index; AST/ALT, aspartic transaminase/alanine transaminase; IL6, Interleukin 6; IL8, Interleukin 8.

We then performed adjusted binary logistic regression analyses to determine the ORs for the relationship of different thyroid parameters with IL-6 and IL-8 (**Table 3**). The results showed that FT3 was negatively associated with IL-6 in Model 1 (OR = 0.503; 95%CI=0.3,0.844; *P*=0.009) and Model 2 (OR=0.529; 95%CI=0.302, 0.926; *P*=0.026), and that TFQI_FT3_ was negatively associated with IL-6 in Model 1 (OR=0.543; 95%CI=0.305, 0.967; *P*=0.038) but not Model 2 (*P*=0.987). FT3/FT4 had no significant association with IL6 in Model 1 (*P*=0.318) or Model 2 (*P*=0.979).

**Table 3:** Binary logistic regression analysis of the relationship of thyroid parameters with IL6 and IL8.

Outcome: IL6≥ 2.38 pg/mL						
	FT3 (per SD pmol/L)		TFQI_FT3_ (per SD)		FT3/FT4 (log, per SD)	
	OR (95%CI)	P	OR (95%CI)	P	OR (95%CI)	P
Model 1^*^	0.503 (0.3,0.844)	0.009	0.543 (0.305,0.967)	0.038	0.729 (0.393,1.354)	0.318
Model 2^†^	0.529 (0.302,0.926)	0.026	1.008 (0.385,2.635)	0.987	1.012 (0.410,2.495)	0.979
Outcome: IL8 ≥ 18.1pg/mL						
	TSH (log, per SD µIU/mL)		TT4RI (log, per SD)		TSHI (log, per SD)	
	OR (95%CI)	P	OR (95%CI)	P	OR (95%CI)	P
Model 1^*^	0.474 (0.277,0.812)	0.007	0.527 (0.311,0.891)	0.017	0.538 (0.287,1.009)	0.053
Model 3^&^	0.343 (0.155,0.759)	0.008	0.398 (0.191,0.831)	0.014	0.531 (0.153,1.721)	0.280

*Model 1 adjusted for sex, Log(age), Log(weight), log (diabetes duration), log (HbA1c), smoking, drinking, hypertension, CHD, use of a RASI, use of a SGLT2I, use of a GLP1RA, and use of a lipid-lowering treatment.

^†^Model 2 adjusted for all Model 1 factors and also for use of metformin, eGFR, 25 (OH)D, Log (UACR), Log (Cys C), Log (TyG index), Log (AST/ALT), and Log (IL-8).

^&^Model 3 adjusted for all Model 1 factors and also for use of metformin, eGFR, 25 (OH)D, Log (UACR), Log (Cys C), Log (TyG index), Log (AST/ALT), and Log (IL6).

TSH, thyroid stimulating hormone; FT3, free triiodothyronine; FT3/FT4, free triiodothyronine/free thyroxine; TSHI, TSH index; TT4RI, TSH T4 resistance index; TFQI_FT3_, the thyroid feedback quantile-based index calculated by FT3.

TSH and TT4RI were negatively associated with IL8in Model 1 (TSH: OR=0.474; 95%CI=0.277,0.812; *P*=0.007; TT4RI: OR=0.527; 95%CI=0.311,0.891; *P*=0.07) and Model 3 (TSH: OR=0.343; 95%CI=0.155, 0.759; *P*=0.008; TT4RI: OR=0.398; 95%CI=0.191, 0.831; *P*=0.014 respectively). TSHI also had a marginally significant negative association with IL-8 in Model 1 (OR=0.538; 95%CI=0.287, 1.009; *P*=0.053), but not Model 3 (*P*=0.280).

## Discussion

The major findings of our cross-sectional study of Chinese patients with euthyroid T2D were that FT3 was negatively associated with IL6, and TSH and TT4RI were negatively associated with IL8 after adjustment for confounding in multiple binary logistic regression models. To our knowledge, this is the first study to explore the association between multiple thyroid parameters, including thyroid hormones and indices of thyroid sensitivity, with markers of chronic inflammation in patients with euthyroid T2D.

Our analysis of the association of thyroid hormones with IL-6 and IL-8 differs from previous research that used simple correlation analysis (4, 5, 15). This is likely because we evaluated these relationships using multiple binary logistic regression models that adjusted for different variables. Thus, our findings of the relationships of these different clinical variables are more robust than the findings of previous studies. In fact, previous studies had some inconsistencies in the reported relationship between thyroid hormones and inflammation markers. For example, a cross-sectional study of T2D patients showed that FT3 was negatively correlated with IL6, supporting our conclusion (4), but this study only used simple correlation analysis and therefore did not exclude the influence of covariates such as HbA1c, eGFR, use of medications, and others (21). In addition, a study of patients with autism and a study of patients with COVID-19 were consistent with our finding of a negative correlation of TSH with IL8 (22). In contrast to our findings, a cross-sectional study of male patients with euthyroid T2D suggested that FT3 was positively associated with IL6 in a simple correlation analysis (5). We attribute the discrepancy of these results with our results to gender differences in the study populations and the lack of adjustment of covariates in the earlier studies.

Several potential mechanisms may explain the association of thyroid hormones with markers of inflammation in T2D. Induction of oxidative stress by hyperglycemia activates NF-κB signaling, and this increases the levels of IL6 and IL8 (10, 11). On one hand, IL6 can inactivate type 1 iodothyronine deiodinase (DIO1), resulting in a decreased production of active tri-iodothyronine from thyroxine (23–25). On the other hand, IL6 enhances the activity of deiodinase 3 (DIO3), thereby accelerating the conversion of FT3 to trans-triiodothyronine and diiodothyronine, which ultimately leads to a reduction in FT3 levels (26). Other research reported that a decreased level of TSH was associated with a higher level of IL8 in individuals with depression, a population with increased risk for diabetes; this relationship may be linked to the activity of DIO1 and to specific variants of phosphodiesterase and the TSH-receptor (27). Future research that investigates the role of deiodinases could contribute to a more comprehensive biological interpretation of our results. For example, previous research has indicated associations of TSH and FT3 with diabetes and related endpoint events (6, 9). Our findings provided novel insights into the underlying mechanisms of these relationships. In particular, within the diabetic population, low levels of FT3 and TSH, even within the normal reference ranges, are significantly correlated with elevated levels of IL6 and IL8.

Thyroid hormone levels alone may not entirely account for the previously reported relationship between the hypothalamus-pituitary-thyroid axis and metabolic disorders (5, 6, 8). A 2009 study first introduced the concept of TSHI, a central thyroid sensitivity index, and reported that approximately 20.9% of individuals with normal TSH and FT4 levels had abnormal TSHI levels (17). Does this abnormality of thyroid hormone sensitivity explain the relationship between metabolic diseases and a disrupted hypothalamus-pituitary-thyroid axis? In fact, numerous recent studies found that thyroid hormone sensitivity was strongly related to metabolic diseases (3, 7, 20, 28–30). Other research found that IL6 and IL8 appeared to play important roles in the development of diabetes and related complications (10–13), and this is consistent with our correlation analysis, which showed that the level of IL6 was associated with a lower eGFR and a longer duration of diabetes. We also focused on the relationship between sensitivity to thyroid hormones and chronic inflammation markers, and found that a lower level of TT4RI was associated with a higher level of IL8, but there was no association between IL6 level and thyroid sensitivity indices after adjustment for confounding. This conclusion was indirectly supported by the results of another study of Chinese patients. This previous large-scale cross-sectional study showed that decreased central thyroid hormone sensitivity was associated with an increased risk for pre-diabetes (28). Similarly, a prospective study of pregnant women found that lower central thyroid sensitivity indices were associated with an increased risk for gestational diabetes (29), and a large multicenter retrospective study of patients with CHD showed that decreased central thyroid sensitivity indices were associated with an increased risk for elevated blood glucose (30).

Several mechanisms have been proposed to explain these relationships. One hypothesis is that a hyperglycemic state may alter the sensitivity of pituitary thyrotrophs due to decreased serum levels of T3 and TSH (31). Furthermore, central thyroid sensitivity may influence leptin secretion (28–30), which can subsequently induce IL8 expression through activation of the leptin receptor (32, 33). Additionally, leptin has can alter the expression of DIO1, ultimately impacting adiposity and glucose metabolism (34). However, a biological explanation for the possibly “protective” effect of lowered central thyroid sensitivity against inflammatory needs further study. Future research endeavors should examine the roles of deiodinases and cytokines, including leptin, ILs, and other cytokines in conjunction with alterations in thyroid hormone levels under conditions of hyperglycemic metabolism. These studies may provide a more comprehensive interpretation of the current findings.

Previous studies have indicated significant associations of 25(OH)D, AST/ALT ratio, duration of diabetes, and other clinical parameters with the progression and outcomes of diabetes (35–37). Consequently, in our evaluation of the relationship between chronic inflammatory markers and thyroid indicators in diabetes, we also performed a preliminary analysis to examine the correlations between these aforementioned clinical indicators and inflammatory markers. Our study identified a negative correlation between IL6 and 25(OH)D. This finding is consistent with prior research. In particular, a study of murine models of T2D demonstrated that vitamin D supplementation effectively reduced levels of inflammatory markers, including IL6 (38). Additionally, a study examining populations affected by COVID-19 indicated that vitamin D supplementation was associated with a decreased level of IL-6 (39). These observations may be attributed to the ability of vitamin D to inhibit the secretion of IL-6 by immune cells (39). Furthermore, our findings indicated a positive correlation between IL-6 and the AST/ALT ratio. This observation aligns with previous research on the association between AST/ALT and diabetic nephropathy (36), suggesting that AST/ALT may be linked to oxidative stress and systemic inflammation. We also demonstrated that the duration of T2D was positively correlated with the IL-6 level. This association is corroborated by prior research that examined patients with type 1 diabetes mellitus (37), and suggests that the progression of diabetes is linked to chronic inflammation.

The strengths of our study are that we thoroughly examined a population of Chinese patients with euthyroid T2D; we characterized multiple thyroid parameters, including TSH, FT3, FT4, TSHI, TF4RI, FT3/FT4 ratio, TFQI_FT3_, and TFQI_FT4_; we measured two indices of chronic inflammation (IL6 and IL8); and we performed a binary logistic regression analysis with adjustment for multiple confounding factors. However, several limitations of our study should be acknowledged. Firstly, although this study was the first to evaluate the association between thyroid sensitivity and chronic inflammation in T2D, our study population was rather small and was limited to a single institution. This prevented the use of subgroup analyses. Nevertheless, the observed associations were sufficiently robust to yield statistically significant results. Secondly, this study utilized a cross-sectional design, limiting our ability to establish causality. Despite adjusting for numerous covariates and controlling for drug-related factors, our lack of data on patient height precluded analysis of the possible influence of BMI on the outcomes. However, previous research indicated that obesity did not significantly impact TSH secretion in individuals with T2D (31), and we tried our best to account for factors such as weight and insulin resistance in the logistic regression analysis. Thirdly, the study population consisted of patients with normal thyroid function, but we lacked data on thyroid antibodies. The presence of thyroid autoimmunity may influence the relationship between thyroid markers and inflammation. Finally, this study employed correlation analyses, and did not examine the underlying mechanisms of relationships. However, a review of the literature indicated that deiodinases may be implicated chronic inflammatory responses (23–26) and are likely to play a critical role in the regulation of thyroid hormones and sensitivity to these hormones. We suggest that future research should focus on elucidating the specific roles of deiodinases in the interplay between thyroid parameters and inflammatory processes. In conclusion, our study of Chinese patients with T2D suggested that thyroid parameters, including thyroid hormones and central thyroid hormone sensitivity, are strongly associated with chronic inflammation, even when these patients have normal thyroid function. Our results provide evidence of a significant interaction between thyroid parameters and the pathophysiology of euthyroid T2D in a Chinese population. Measurement of these thyroid parameters may facilitate the monitoring and evaluation of novel diabetes treatments that target chronic inflammation. Additionally, these parameters may be useful as predictors for endpoint events associated with inflammatory processes.

## Data Availability

The raw data supporting the conclusions of this article will be made available by the authors, without undue reservation.
